# Explaining the unexplainable: designing a national strategy on classroom communication concerning the 22 July terror attack in Norway

**DOI:** 10.3402/ejpt.v5.22758

**Published:** 2014-07-02

**Authors:** Jon-Håkon Schultz, Åse Langballe, Magne Raundalen

**Affiliations:** 1 Norwegian Centre for Violence and Traumatic Stress Studies, Oslo, Norway; 2 Department of Education, University of Tromsø, Tromsø, Norway; 3 Center for Crisis Psychology, Bergen, Norway

**Keywords:** Teacher role, school-based interventions, crisis and terror

## Abstract

**Background:**

In the context of crisis and disasters, school-aged children are a vulnerable group with fewer coping resources than adults. The school is a key arena for preventive interventions; teachers can be given a key role in large-scale school-based interventions following a man-made or natural disaster.

**Objectives:**

This paper describes a practical example of designing a school-based population-level intervention.

**Methods:**

The preventive measures were delivered as a national communication strategy between teachers and pupils aged 6–19 concerning the terror attack on 22 July 2011 in Norway. The strategy is based on principles from international research.

**Results:**

The presentation contributes to the discussion of defining the teacher's role in school-based crisis interventions and dealing with high-intensity media coverage of war, terror, and catastrophes.

**Conclusions:**

The presentation provides educational and psychological perspectives on how teachers can take an active role in helping pupils to deal with such events through two approaches: the therapeutic approach, to restore calm and feelings of safety; and the educational approach, to foster reflection and deeper understanding.

Studies on the consequences of disasters and terrorism in the general population are limited (Miguel-Tobal et al., 2006; Norris, Friedman, & Watson, 2002a, b). Studies indicate that psychological health and social relations can be affected within the population in general, but especially for populations living geographically close to the attack. Both the general population in New York after the September 11, 2001, attack and the population in Madrid after the Madrid bombing in 2004 showed a two-fold increase in the level of depression over the following months (Miguel-Tobal et al., 2006). In the days immediately following the 9/11 attacks, a US national sample of adults reported that 35% of their children showed one or more stress symptoms and 47% of the children were worrying about their own safety or the safety of loved ones (Schuster et al., 2001). A high proportion (28.6%) of New York City public school pupils showed one mental disorder 6 months after the attack, with a higher prevalence among pupils living in close proximity to the World Trade Center site. The data indicate a relationship between the level of exposure and child anxiety and depression disorders at the community level (Hoven et al., 2005).

Several studies have replicated findings that television exposure of catastrophes is associated with stress reactions among children, young adults, and adults. In the Oklahoma City bombing, television exposure was directly related to posttraumatic stress symptomatology measured at 7 weeks among middle school students (Pfefferbaum et al., 2001). Three months after the 9/11 attacks, elementary school children in Washington, DC, had high levels of psychological reactions that were best understood in the context of their indirect exposure, primarily through television news (Philps, Prince, & Schiebelhut, 2004).

Reviewing 60,000 disaster victims from 160 samples, Norris et al. (2002a) showed that school-aged youths are more likely than adults to be impaired, and more severely affected, by disasters. This indicates that children and youths seem, on average, to be less well equipped than adults to cope with disasters. The cognitive abilities and lack of life experience of school children may reduce their ability to, for example, handle acute helplessness, or comprehend and make sense of the world; and may cause a loss in perceived safety and social support. Another central finding from the meta-analysis study by Norris and colleagues (2002a) is that being exposed to mass violence, such as terrorism and shooting sprees, was far more likely to result in a higher symptom rate than exposure to natural and technological disasters.

On 22 July 2011, a car bomb exploded outside the main government building in Norway. Eight people were killed and more than 100 were injured. The perpetrator then engaged in a mass killing at Utøya, where the youth organisation of the Labour Party was holding its annual summer camp attended by 564 mostly young people. Sixty-nine were killed, many were injured, and 56 were hospitalised.

Several studies have investigated the social and emotional responses in the general Norwegian population following the terrorist attacks. The first study (Wollebæk, Enjolras, Kari Steen-Johnsen, & Ødegård, 2012) indicated that Norwegians in general had not become less trusting. The exception was found among one-third of the young population (18–24 years) who expressed lower levels of interpersonal trust. The second study (Thoresen, Aakvaag, Wentzel-Larsen, Dyb, & Hjemdal, 2012) showed that strong emotional responses were experienced immediately after the attacks among young adults and adults. The third study (Nordanger et al., 2013) focused on secondary school students aged between 17 and 19. As many as 55.7% experienced the events as threatening “to some extent” to their own lives or the lives of those close to them. The three studies indicate strong immediate reactions among the general adult and adolescent populations. Less is known about the reactions of those under 16, how they dealt with the terrorist attacks, and their level of media exposure. The Norwegian media covered the 22 July terrorist events by featuring a constant flow of interviews with survivors and family members of those who had lost their lives. The interviews revealed strong feelings and grotesque details. During the weekend after the terrorist attacks, respondents reported spending an extensive amount of time watching the news: a mean total of 17 hours in Oslo, and 16 elsewhere in Norway (Thoresen et al., 2012).

## Identifying characteristics of the Norwegian terror attack

To determine the relevant preventive measures for indirectly affected school-aged children and youths, we took the following points into account, based on previous research and the specifics of the current terrorist attack and mass killings: 1) exposure to man-made disasters, such as terror attacks and shooting sprees, tends to cause more severe symptoms than exposure to natural disasters; 2) indirect exposure can cause psychological distress, such as anxiety and depression, and impact upon perception and behaviour both in the short and long term; 3) in the context of disasters, school-aged children may be an especially vulnerable group with less coping resources than adults; 4) high intensity media coverage may cause indirect exposure; 5) Norway was affected as a nation; the political leaders were targeted, as well as youths at the summer camp that geographically represented most parts of Norway; and 6) youths were targeted in the attack, causing a potential high grade of identification among the same age group.

## Designing a population-level intervention

Immediately after the 22 July attack, contact was established between the Ministry of Education, the Directorate of Education, and the Norwegian Centre for Violence and Traumatic Stress Studies. A mandate was negotiated that called for a national strategy on classroom communication, aimed at restoring a sense of security among pupils. The intervention primarily targeted pupils aged 6–16 and secondarily pupils aged 17–19, covering primary, lower secondary, and upper secondary school.

In order to provide advice based on current needs among indirectly affected pupils, a series of interviews was arranged. Over a 10-month period, teachers, school administrators, and pupils were interviewed. The content of these interviews was discussed by groups of researchers and practitioners. Group interviews with 36 teachers and school leaders in primary and lower secondary school indicated uncertainty on how teachers should engage in conversations about the terror attack. The majority argued that teachers should address the terror in class, at the same time they were uncertain whether this could evoke fear among the pupils. Out of individual interviews with 54 pupils aged 6, 7, and 8 years, 51 could relate detailed narratives about the terrorist attack and the mass killings (Jørgensen, 2013). Nineteen of the children gave narratives that had elements of fiction, confusion, and misunderstanding; such elements contributed further to the children's fears. Seven months after the attacks, 20 of the 54 children reported specific fear of the perpetrator and that something similar would happen to them or their family.

### A national strategy on classroom communication

The Minister of Education held a press conference and gave several high impact interviews prior to the start of the school year to present the scope of the school-based intervention. She gave teachers the clear task of “restoring pupils’ feelings of security.” The school year was to start with a remembrance ceremony for the deceased; this would serve as the starting point for on-going communication between teachers and pupils.

Assuming Norwegian teachers in general were uncertain regarding the teacher's role in dealing with crises, we decided upon a strategy with a step-by-step procedure with suggestions regarding practical explanations to assist pupils in their meaning making. Several studies have shown that using a highly structured protocol is effective in changing professional behaviour in a desired direction (Lamb, Sternberg, Orbach, Hershkowitz, & Esplin, 2000). Suggestions on how to provide explanations and how to engage in classroom communication were written in full text as model language. The communication strategy as a whole was outlined in two separate documents that were adopted as a national strategy (Raundalen, Schultz, & Langballe, 2012; Schultz & Raundalen, 2011).

The first document, *Educational First Aid* (Schultz & Raundalen, 2011) was sent from central school authorities to all Norwegian schools, together with an official letter, stating the urgency and need for the school-based intervention. This document focused on clarifying the teacher's role in helping pupils of various ages to feel safe and secure, through a therapeutic perspective and an educational perspective. The aim was to provide teachers with a context for Antonovsky's theory (1987) related to establishing a sense of coherence and making a crisis event intelligible, manageable, and meaningful. This document was further inspired by the five empirically supported principles of intervention and prevention efforts for the early to midterm stages of crisis (Hobfoll et al., 2007) and the concept of psychological first aid (Brymer et al., 2006). The goal was to empower teachers to be proactive and engage in classroom communication. Below is a quote from the paragraph where teachers are guided towards providing an explanation, and invite pupils to participate in a conversation, about why and how the perpetrator could carry out such atrocities:

*Why did he do it?* We are aware that many teachers for obvious reasons would want to avoid focusing on this question. To the children, however, this is the key question, because it is crucial to the new sense of safety they are trying to establish: that this will not happen again. (…).

The second document (Raundalen, Schultz, & Langballe, 2012) was circulated prior to the trial: *Suggestions for how teachers can protect students*. The document asked teachers to prepare their pupils for weeks of high-intensity media coverage full of grotesque and frightening details. They were asked to prepare a pedagogical plan; the document offered practical suggestions on what such a plan could entail. There had been a heated and lengthy debate in the national media regarding whether the perpetrator should be diagnosed as sane or insane – with consequences of being sentenced to jail or for hospital treatment. Suggestions with model language on how to explain this to pupils were given in full text. In both documents there were also presentations on the mental, psychological, and sociological factors often used to explain how a human being can carry out mass murder. In other words, these documents sought to provide explanations for the unexplainable, to support teachers in their work of helping pupils to comprehend and understand. The two examples below with model language are from the second document.

*What does the perpetrator himself think?* The perpetrator has claimed all along that he is sane and knew what he was doing. He planned everything in detail, and tricked the youths on Utøya in a horrible way by dressing up as a policeman and saying that he would protect them. Nevertheless, you can say that it was completely sick – what he did and what he writes and says seems absolutely sick. We already know that normal people can also do absolutely sick things. Therefore it will be up to the experts and the court to determine whether the perpetrator has a mental illness which means that he will need to be taken care of in a hospital in the future.

*The most important things:* It is important that we do not spend more time than necessary on these questions and that we gradually try to put them behind us and carry on with normal things in our lives. The most important thing for all of us in the future is that those who were injured receive our help, support, and respect, and that the same applies to all of those who are grieving over the great loss of human life. Many of those who were not physically injured when they were in the Government Complex or on Utøya must also be given help and support to get over their horrible memories and to get on with their lives at school, their studies, or their jobs.

### Therapeutic and educational perspectives on classroom communication

The therapeutic perspective focused on offering reassurance when new and frightening news was broadcast, and on restoring feelings of security among the general student body with a special focus on potentially vulnerable groups, such as refugees from war-affected countries, and pupils who had experienced domestic violence. An especially vulnerable group comprised all pupils with a Muslim background because the perpetrator had named this group as the ideological reason for attacking Norwegian politicians. The therapeutic perspective also included participating in rituals for remembering the deceased, dealing with sorrow, and strengthening self-efficacy and community bonds. When formal classroom instruction is added to the action of participating in school and community rituals, the therapeutic and the educational perspective are combined. Building self-efficacy and community-efficacy becomes an educational goal when rituals are put on the educational agenda, and are practiced and explained.

In our interviews with teachers and pupils the following questions were frequently raised by pupils: Will he get out of prison? Are we safe now? Why did he do it? How will the survivors cope with the crisis? In order to deal actively with such questions, teachers were advised to allow for classroom communication where pupils could ask questions, present their thoughts, and receive new information from their teacher. Based on this communication teachers were advised to follow up by helping pupils to understand: 1) how the health care system works; how victims react and how they are being helped, 2) how the media functions in on-going crises, 3) how the judicial system works, 4) the development of psychiatric diseases, and 5) radicalisation processes. Teaching in these topics is within an educational perspective and provides a possible learning outcome of understanding – allowing the pupils to discuss and formulate questions in order to make the crisis event manageable.

## Experiences, challenges, and suggestions

After the terrorist attack and the mass murders of 2011, it was our assumption, based on previous research, that a significant proportion of Norwegian pupils would be indirectly affected. The school-based population-level intervention was a preventive measure against stress reactions and a potential reduction in learning capacity. The intervention was intended to improve understanding of terrorism, society, stress reactions, and how to deal with sorrow and sadness. The teacher was chosen as the implementer because of the closeness shared between teachers and pupils and because the goals of the intervention were considered to fall within the teacher role. In the absence of a systematic evaluation of the intervention we refer to feedback during the implementation process indicating a somewhat mixed outcome. The intervention was widely used, it empowered teachers to engage in classroom conversations and clarified their role – but we also registered a marked response from teachers meaning such communication was not appropriate in primary school because of the possibility of evoking fear.

In the aftermath of a man-made or natural disaster, a large-scale school-based intervention appears important because it allows for a cost-effective, rapidly deployed, and far reaching intervention targeting school children as a vulnerable group. The structure of the described intervention is adapted for the school environment, empowering teachers to play the key role in crisis management within their own arena. We argue that, in general, teachers ought to take a proactive role in crises with high-intensity media coverage. Two perspectives can define their role: the therapeutic perspective, where the pupils are calmed and their feelings of safety restored; and the educational perspective, looking for and defining the learning potential imbedded in the crisis, with the goal of fostering reflection and deeper understanding.

To avoid relying on chance and individual initiative for determining what kind of help and support pupils receive at school for dealing with their feelings and understanding major conflicts and catastrophes, media-intensive crises should be put on the schools pedagogical agenda. If teachers are to work on including crises in their curriculum, they need a clear mandate from the school authorities regarding the role and responsibility of the schools in educating pupils about international and national crises. Having a previously established relationship between school authorities and professionals who design the intervention is time effective. In this current case it proved essential to conduct interviews with pupils of different ages, teachers, and school leaders, in order to inform the nature of the intervention in a fluid crisis situation. It was also necessary for the developers of the intervention to have a close relationship with the school authorities and to possess the intervention mandate for a considerable amount of time; in this case, for 10 months.
